# Overweight in the Elderly Induces a Switch in Energy Metabolism that Undermines Muscle Integrity

**DOI:** 10.14336/AD.2018.0430

**Published:** 2019-04-01

**Authors:** Yaiza Potes, Zulema Pérez-Martinez, Juan C. Bermejo-Millo, Adrian Rubio-Gonzalez, María Fernandez-Fernández, Manuel Bermudez, Jose M. Arche, Juan J. Solano, Jose A. Boga, Mamen Oliván, Beatriz Caballero, Ignacio Vega-Naredo, Ana Coto-Montes

**Affiliations:** ^1^Department of Morphology and Cell Biology, Faculty of Medicine, University of Oviedo, Asturias, Spain; ^2^Instituto de Investigación Sanitaria del Principado de Asturias (ISPA), Spain; ^3^Microbiology Service, Central University Hospital of Asturias, Asturias, Spain; ^4^Geriatric Service, Monte Naranco Hospital, Asturias, Spain; ^5^Servicio Regional de Investigación y Desarrollo Agroalimentario (SERIDA), Asturias, Spain; ^5^Instituto de Investigación Sanitaria del Principado de Asturias (ISPA), Spain

**Keywords:** overweight, elderly, glycolysis, mitochondrial metabolism, aged-related atrophy

## Abstract

Aging is characterized by a progressive loss of skeletal muscle mass and function (sarcopenia). Obesity exacerbates age-related decline and lead to frailty. Skeletal muscle fat infiltration increases with aging and seems to be crucial for the progression of sarcopenia. Additionally, skeletal muscle plasticity modulates metabolic adaptation to different pathophysiological situations. Thus, cellular bioenergetics and mitochondrial profile were studied in the skeletal muscle of overweight aged people without reaching obesity to prevent this extreme situation. Overweight aged muscle lacked ATP production, as indicated by defects in the phosphagen system, glycolysis and especially mostly by oxidative phosphorylation metabolic pathway. Overweight subjects exhibited an inhibition of mitophagy that was linked to an increase in mitochondrial biogenesis that underlies the accumulation of dysfunctional mitochondria and encourages the onset of sarcopenia. As a strategy to maintain cellular homeostasis, overweight subjects experienced a metabolic switch from oxidative to lactic acid fermentation metabolism, which allows continued ATP production under mitochondrial dysfunction, but without reaching physiological aged basal levels. This ATP depletion induced early signs of impaired contractile function and a decline in skeletal muscle structural integrity, evidenced by lower levels of filamin C. Our findings reveal the main effector pathways at an early stage of obesity and highlight the importance of mitochondrial metabolism in overweight and obese individuals. Exploiting mitochondrial profiles for therapeutic purposes in humans is an ambitious strategy for treating muscle impairment diseases.

Sarcopenia, an age-related degenerative loss of skeletal muscle mass, quality and strength, causes a functional decline in the elderly. This decline initiates around age 40, and by the 8^th^ decade of life, it results in a loss of up to 50% of muscle mass [1]. Sarcopenia is widely considered a multifactorial pathology that affects the neuromuscular junction, hormone production, sensitivity of hormone receptors, bone mineral density and, finally, inducing physical disability [2]. Furthermore, patients with hip fractures exhibit a higher prevalence of sarcopenia [3]. The mechanisms underlying loss of muscle quality with age are complex, but several studies have described a maldistribution of fat in old age driving the accumulation of lipids in muscle tissue [4, 5]. The deleterious effect of obesity and the excess of fat infiltration can affect muscle satellite cells by reducing number and function [6, 7], ultimately compromising the differentiation capacity and increasing the risk for death in sarcopenic situations [8]. Given that the resting stem cells of geriatric skeletal muscle lose quiescence by switching to an irreversible senescence state [9], the lipotoxicity of lipid accumulation in the skeletal muscle tissue of elderly has recently gained attention as a potential contributor to frailty and muscle wasting.

Skeletal muscle has high-energy demand and alterations in cellular bioenergetics is linked to wide variety of pathological conditions such as aging and obesity causing muscle wasting [10, 11]. This is due directly to the fact the energy metabolism can influence the maintenance of protein balance and skeletal muscle mass, as well as the regulation of cell-fate and the differentiation of stem cells [12-14]. Similarly, there is a potential impact of oxidized proteins on the altered metabolism of senescent satellite cells [15]. Therefore, mitochondria, as the main energy hub of the cell and the main intracellular source of reactive oxygen species (ROS), are crucial for muscle mass maintenance. Mitochondria are organelles with high dynamic plasticity displayed by fission and fusion mechanisms to adapt rapidly in response to pathological situations [16]. However, impaired mitochondrial function and unbalance remodeling of mitochondrial network morphology controlled by a machinery of pro-fusion and fission proteins, are considered primary instigators of atrophy [17]. A balance between mitochondrial biogenesis and mitochondrial selective autophagy (mitophagy) is also essential for maintaining the cellular metabolic state. Thus, mitochondrial function plays an essential role in aging, particularly in the skeletal muscle, since a shift in energy production anticipates the onset of sarcopenia [10]. This could be mainly due to mitochondrial bioenergetics and mitochondrial dynamic maintenance that are essential in the regulation of calcium oscillations supporting the excitation-contraction coupling mechanism in skeletal muscle [18]. Moreover, altered mitochondrial energy metabolism targets ROS production that could affect Ryanodine receptor 1 (RYR1) channel. RYR1 is vital for coupling the process of excitation and contraction and for skeletal muscle calcium homeostasis. Under stressors, the RYR1 channel experiences a complex remodeling based on phosphorylation that results in leaky channels, which causes a massive release of Ca^2+^ [19]. Hwang and colleagues also showed a downregulation of mitochondrial function in obesity [20], so excess adipose tissue in aging populations could be of paramount importance for developing mitochondrial metabolic impairment and the progression of sarcopenia.

Despite obesity prevention strategies and efforts to develop treatments, the obese population has more than doubled over the last three decades (Obesity and overweight; www.who.int/mediacentre/factsheets/fs311/en/). This is especially relevant in older adults who experience a progressive increase of muscle fat infiltration with aging that may be a central aspect of sarcopenia. Given obesity is related to metabolic alterations and functional dependence in the elderly population represents a global public health challenge to promote healthy aging. Moreover, the study of overweight is beginning to attract attention to combat obesity-associated alterations and could have a major impact on effectiveness of prevention efforts, especially in aging. Taking into account that energy metabolism is poorly characterized in sarcopenic obesity, the main objective of the present work was to study cellular bioenergetics and mitochondrial quality control mechanisms. Both have a direct effect on muscle contraction and integrity in the skeletal muscle of overweight older adults. This study is essential to identify potential therapeutic targets at an early stage of obesity.

## MATERIALS AND METHODS

### Participants

The study population (N=39) comprised randomly selected female and male patients from the HIPA cohort (N=131) (hip fracture in older patients undergoing surgery in the region of Asturias (Spain)). The number of female patients recruited was much higher, however overweight has not been influenced by the impact of sex at muscle level. Thus, both genders were included and homogeneously distributed in the both experimental groups. All participants were informed about the purposes, procedures, risks and benefits of the study by experienced geriatricians and provided valid signed informed consent. Geriatricians also carried out the initial evaluations, which included an exhaustive review of medical and pharmacological histories and an assessment of the functional abilities and comorbid conditions of the subjects using the Barthel and Charlson indexes, respectively. Both questionnaires have been previously validated and their use demonstrated in an elderly Spanish population [21, 22]. Design and methodology are fully described in a previous publication [23]. The inclusion criteria were age ≥ 70 years and Barthel Index ≥ 90. The exclusion criteria were high comorbidity (Charlson index ≥ 2), cognitive impairment, obesity (body mass index (BMI) > 30), oncological hip fractures and terminal illnesses. Elderly patients were functionally independent and healthy without any risk to have one or more additional disorders aside from the hip fracture. Participants did not significantly engage in any physical activity.

The BMI (Kg/m^2^) ratified by the World Health Organization (Body Mass Index classification; URL: http://apps.who.int/bmi/index.jsp?introPage=intro_3.html) was used to divide an elderly population (85±6 years) into two balanced groups. Twenty-one normal-weight and eighteen overweight patients (22.4±1.9 and 28.0±1.7 BMI, respectively) were recruited and hospitalized due to hip fracture during emergency service at the Monte Naranco Hospital (Oviedo, Spain). The Clinical Research Committee of the Central University Hospital of Asturias (HUCA) approved this work. The study complied with the Declaration of Helsinki.

### Muscle collection

Skeletal muscle biopsies of the *Vastus lateralis* were taken during hip surgery and immediately frozen at -80°C until further use. Muscles (0.2 g) from each patient were homogenized using an Ultra-turrax homogenizer (Ultra-Turrax T25 digital; IKA, Staufen, Germany) at 4°C in 1 mL of lysis buffer (50 mM phosphate buffer, pH 7.5, 1 mM NaF, 1 mM Na_3_VO_4_, 1 mM PMSF, 0.1% Triton-X 100) and centrifuged for 6 min at 1500 g and 4°C. The supernatants were collected and the amount of protein was measured using the Bradford method [24].

### Peptide mass fingerprinting

Aliquots of muscle homogenate (25 μg per sample) were solubilized in Laemmli sample buffer (BioRad Laboratories, Inc., CA, USA), denatured by boiling at 100°C for 5 min and separated by SDS-PAGE. Molecular weight standards (Precision Plus Protein All Blue Standards, BioRad) were also run on each gel to identify the proteins molecular weights. One-dimensional gels were stained by Coomassie Brilliant Blue R-250 (BioRad), and the stained gel images were semiquantitatively analyzed using the Image Studio Lite 3.1.4 software for Macintosh (LI-COR Biosciences, NE, USA).

Bands of interest were manually excised and sent for identification by peptide mass fingerprint to the Inbiotec S.L. (León, Spain) proteomics laboratory, where the samples were processed and analyzed with a 4800 Proteomics Analyzer matrix-assisted laser desorption ionization time-of-flight (MALDI-TOF/TOF) mass spectrometer (ABSciex, MA, USA) according to the previously described methods of Oliván et al [25]. A database search on Mascot Generic Files combining MS and MS/MS spectra was performed using Mascot v 2.2 from Matrix Science through the Global Protein Server v 3.6 (ABSciex). When the Mascot score was greater than 85 points, the identified protein was considered a valid candidate.

### Western blot immunoassays

Western blot immunoassays of the human skeletal muscle samples were performed according to the instructions previously described by our research group [26] using the following primary antibodies with their respective dilution: BNIP3 (1:1000) (3769, Cell Signaling, Danvers, MA, USA); CaMKII (1:500) (3362, Cell Signaling); CI-20 (1:10000) (NDUFB8) (ab110242, Abcam, Cambridge, UK); CII-30 (1:10000) (SDHB) (ab14714, Abcam); CIII-Core II (1:10000) (UQCRC2) (ab14745, Abcam); CIV-I (MTCO1) (1:10000) (ab14705, Abcam); CV-a (ATP5A) (1:10000) (ab14748, Abcam); Cyclophilin D (1:5000) (ab110324 (MSA04), Abcam); DRP1 (1:250) (D6C7) (8570S, Cell Signaling); FIS1 (1:500) (sc-48865, Santa Cruz Biotechnology, Santa Cruz, CA, USA); MFN2 (1:500) (D2D10) (9482S, Cell Signaling); NIX (1:1000) (N0399, Sigma-Aldrich, MO, USA); OPA1 (1:200) (sc-30573, Santa Cruz Biotechnology); phospho-CaMKII (1:500) (3361, Cell Signaling); Phospho-Pyruvate Dehydrogenase (1:1000) (ab92696, Abcam); Pyruvate Dehydrogenase (1:1000) (ab110330, Abcam) phospho-RYR1 (1:2000) (ab59225, Abcam) and TOM20 (1:1000) (42406, Cell Signaling).

Digital images were analyzed quantitatively using the Image Studio Lite 3.1.4 software. Variations in the levels of typical housekeeping proteins (GAPDH, β-actin and α-tubulin) were found, so the results were normalized to Ponceau S staining as a loading control [27]. Analyses of the mitochondrial proteins were normalized to TOM20 to express the results as a function of mitochondrial mass for each condition.

### RNA extraction and RT-qPCR analysis

Total human muscle tissue RNA was extracted using the TRI reagent (T9424, Sigma-Aldrich) and quantified using NANO DROP 2000 (Thermo Fisher Scientific, MA, USA). Complementary DNA (cDNA) was synthesized using the High Capacity cDNA reverse transcription Kit (4368814, Applied Biosystems, CA, USA) following the manufacturer’s protocols. Gene expression was analyzed by quantitative real-time PCR (RT-qPCR) assays using a StepOne real-time PCR system (Applied Biosystems) and the appropriate primers according to the Power SYBR Green PCR Master Mix protocol (4367659, Applied Biosystems). Primer pairs used for RT-PCR were for *ACACA* , forward: 5’-AATGGCATTGCAGCAGTGAA-3’, reverse: 5’-CACATAGTGATCTGCCATCTTAAT GTATT-3’; *ADIPOR1* , forward: 5’-CCCACCATGCC ATGGAGA-3’, reverse: 5’-GCCATGTAGCAGGTAG TCGTTGT-3’; *ADIPOR2* , forward: 5’-CAGGAAGA TGAGGGCTTTATGG-3’, reverse: 5’-GAGGAAGTCA TTATCCTTGAGCCA-3’; *CALSTABIN* , forward: 5’-GGGGATGCTTGAAGATGGAA-3’, reverse: 5’-TTG GCTCTCTGACCCACACTC-3’; *DRP1* , forward: 5’-CATATTTTCTCATTGTCAGAAAGAATATTCA-3’, reverse: 5’-5’-CATCCAATAAGGATGATTTATA CAG CTG-3’; *MFN2* , forward: 5’-AACAGGTTCTGGA CGTCAAAGG-3’, reverse: 5’-GGCATTGATCACG GTGCTCT-3’; *PPARGC1A* , forward: 5’-GACTTGGAT ACAGACAGCTTTCTGG-3’, reverse: 5’-GCTAGCAA GTTTGCCTCATTCTCT-3’; *PPARA* , forward: 5’-TGAAGAACTTCAACATGAACAAG-3’, reverse: 5’-TTGGCCACCAGCGTCTTC-3’; *RYR1* , forward: 5’-AAGGCGAAGACGAGGTCCA-3’, reverse: 5’-TTCT GCGCGTTGCTGTGG-3’; *SERCA1* , forward: 5’-CTGA CCGCAAGTCAGTGCAA-3’, reverse: 5’-GGATGG ACTGGTCAACCCG-3’; *TBP* , forward: 5’-TGCACA GGAGCCAAGAGTGAA- 3’, reverse: 5’-CACATCA CAGCTCCCCACCA- 3’. The amplification protocol included the following stages: a holding stage for 10 min at 95 °C; a cycling stage for 40 cycles of 15 s at 95 °C and 1 min at 60 °C; and finally, a melt curve stage for 15 s at 95 °C, 1 min at 60°C and 15 min at 95 °C. cDNA samples were run, and the average cycle threshold (Ct) value at which each gene was detectable was calculated. The Ct of the TATA-binding protein (TBP) was used for normalization. The relative mRNA expression levels were calculated using the 2^-ΔΔCt^ method [28].

### DNA extraction and mitochondrial DNA content determination

Total DNA from human muscle tissue homogenate was isolated using MagNA Pure LC Total Nucleic Acid Isolation Kit (03038505001, Roche Diagnostics, Berlin, Germnay) according to manufacturer’s recommended protocol. NANO DROP 2000 was used to quantify DNA abundance and purity. RT-qPCR using a StepOne real-time PCR system was performed according to the Power SYBR Green PCR Master Mix instructions and the amplification protocol previously described. Primers pairs used were tRNA Leu(UUR), forward: 5’-CACCCA AGAACAGGGTTTGT-3’, reverse: 5’-TGGCCAT GGGTATGTTGTTA-3’ for mtDNA determination and β2-microglobulin, forward: 5’-TGCTGTCTCCATGTT TGATGTATCT-3’, reverse: 5’-TCTCTGCTCCCCACC TCTAAGT-3’ for genomic DNA determination [29]. mtDNA content was compared against genomic DNA and was calculated using Venegas and Halberg method [29].

### ATP levels

The determination of ATP levels was assessed in homogenized muscle tissue using the Adenosine 5’-triphosphate (ATP) Bioluminescent Assay Kit (FLAA, Sigma-Aldrich). This assay is based on the consumption of ATP when firefly luciferase catalyzes the oxidation of D-luciferin. The ATP content was determined by the measure of the light emission with a luminometer. The concentrations of ATP are expressed as nmol ATP/g protein.

### Blood collection and lactate concentration

Venous blood samples were taken by venipuncture before 10:00 AM to preclude circadian variation. Blood samples were drawn into plasma preparation vacutainer tubes containing EDTA (BD, NJ, USA). After processing, the plasma was divided into aliquots and stored at -80°C as previously described [30-32]. One milliliter of plasma was sent to the laboratory of the San Rafael Hospital (Madrid, Spain) for the determination of lactate production. The lactate production was analyzed by a colorimetric method based on lactate oxidation by two sequential enzymatic reactions: lactate oxidase and HRP using the analyzer VITROS FUSION 5.1 (Ortho Clinical Diagnostics, NJ, USA). Data are expressed as mg/dL, and the lactate normal range is 5.7-20.

### Pyruvate kinase activity

Pyruvate kinase activity was determined in skeletal muscle tissue using a commercially available Pyruvate Kinase Activity Assay Kit (MAK072, Sigma-Aldrich), which measures the increase in pyruvate content per minute. This is based on the reaction of pyruvate kinase enzyme that catalyzes the transfer of a phosphate group from phosphoenolpyruvate (PEP) to ADP, yielding one molecule of pyruvate and ATP. Results are expressed as nmol pyruvate/minute and mg protein.

### Protein oxidative damage

The carbonyl content in oxidatively modified proteins of skeletal muscle tissue was determined following Levine et al. protocol [33] with modifications described by Coto-Montes and Hardeland [34]. The method is based on the reaction of 2,4-dinitrophenylhydrazine with the carbonyl groups of damaged proteins. Data are expressed as nmol of protein carbonyl/mg of protein.

### Statistical analysis

The statistical software package GraphPad Prism 6.0 for Macintosh (GraphPad Software, Inc. CA, USA) was used for all statistical analyses. Data are mean values ± standard error of the mean (SEM). The normality of the data was analyzed using the Kolmogorov-Smirnov test. Comparisons between both experimental groups were carried out using Student’s T-test or the Mann-Whitney U test for continuous variables. A p<0.05 was considered statistically significant.

## RESULTS

### Subject characteristics

The characteristics of participants have been described in a previous publication [26] and are summarized in Supplementary Table 1. In overweight patients, the BMI, abdominal and pelvic perimeters were significantly higher compared to those of the lean subjects (p<0.0001). The other two variables, the Barthel and Charlson indexes, which consider independence measures and low-grade comorbidity, respectively, did not have significant differences between both groups.

### Electrophoretic pattern

SDS-PAGE separation and staining of gels for skeletal muscle proteins from normal and overweight subjects (see Supplementary Fig. 1) allowed the extraction of proteins of interest and their subsequent identification by MS/MS MALDI-TOFF analysis.

**Figure 1.**
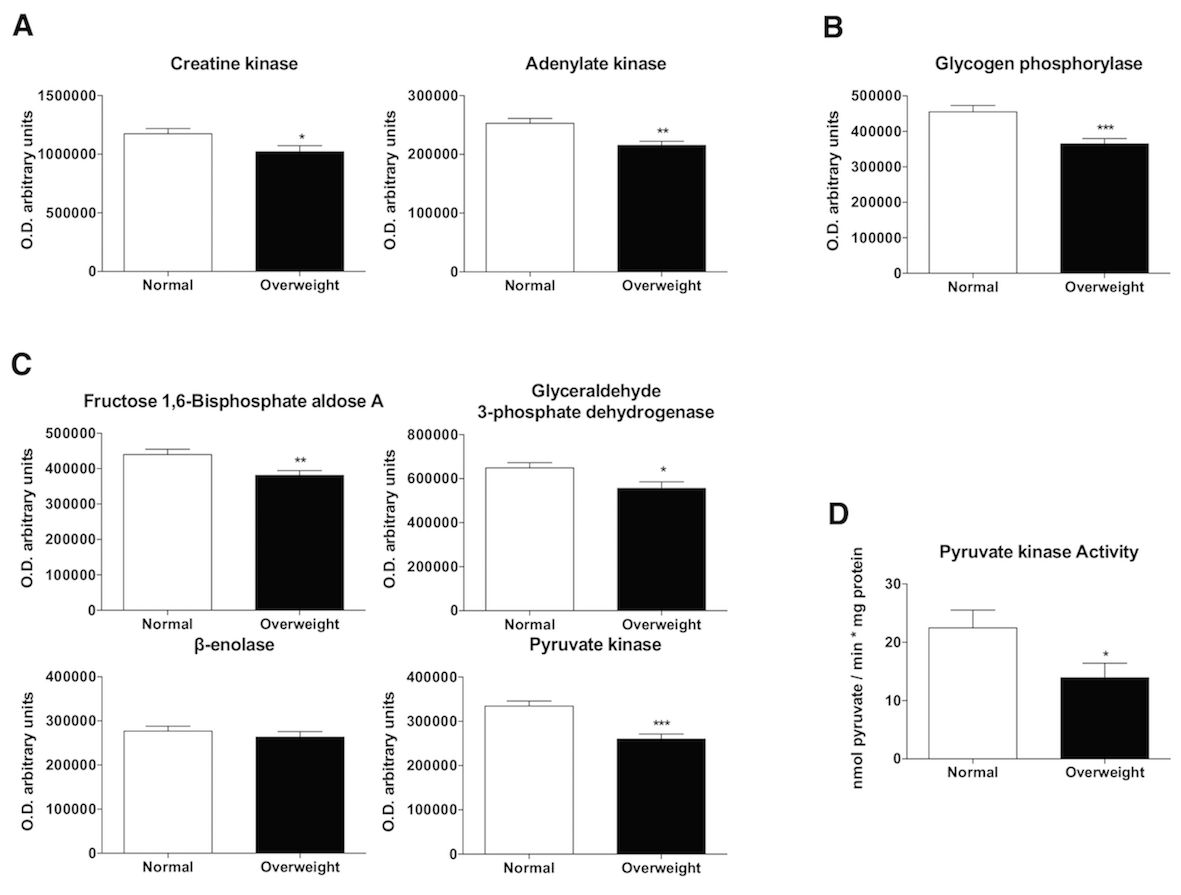
Phosphagen pathway, glycogenolysis and glycolysis. Laser desorption/ionization-time of flight (MALDI-TOF/TOF) mass spectrometry analysis for studying the phosphagen system, glycogenolysis and glycolysis pathways in muscle tissues of normal weight and overweight patients. (A) Levels of proteins involved in the phosphagen system (creatine kinase and adenylate kinase). Bar charts show the means of semi-quantitative optical density (O.D.) ± SEM. (B) Levels of the main protein involved in the regulation of glycogenolysis (glycogen phosphorylase). Bar charts show the means of semi-quantitative optical density (O.D.) ± SEM. (C) Levels of proteins involved in glycolysis (fructose 1,6-bisphosphate aldolase A, glyceraldehyde 3-phosphate dehydrogenase, β-enolase and pyruvate kinase). Bar charts show the means of semi-quantitative optical density (O.D.) ± SEM. (D) Pyruvate kinase activity determination as the main enzyme involved in glycolysis regulation. The pyruvate kinase activity is presented as nmol pyruvate / min*mg protein. Data are expressed as the mean ± SEM. *, P < 0.05; **, P < 0.01; ***, P < 0.001.

**Figure 2.**
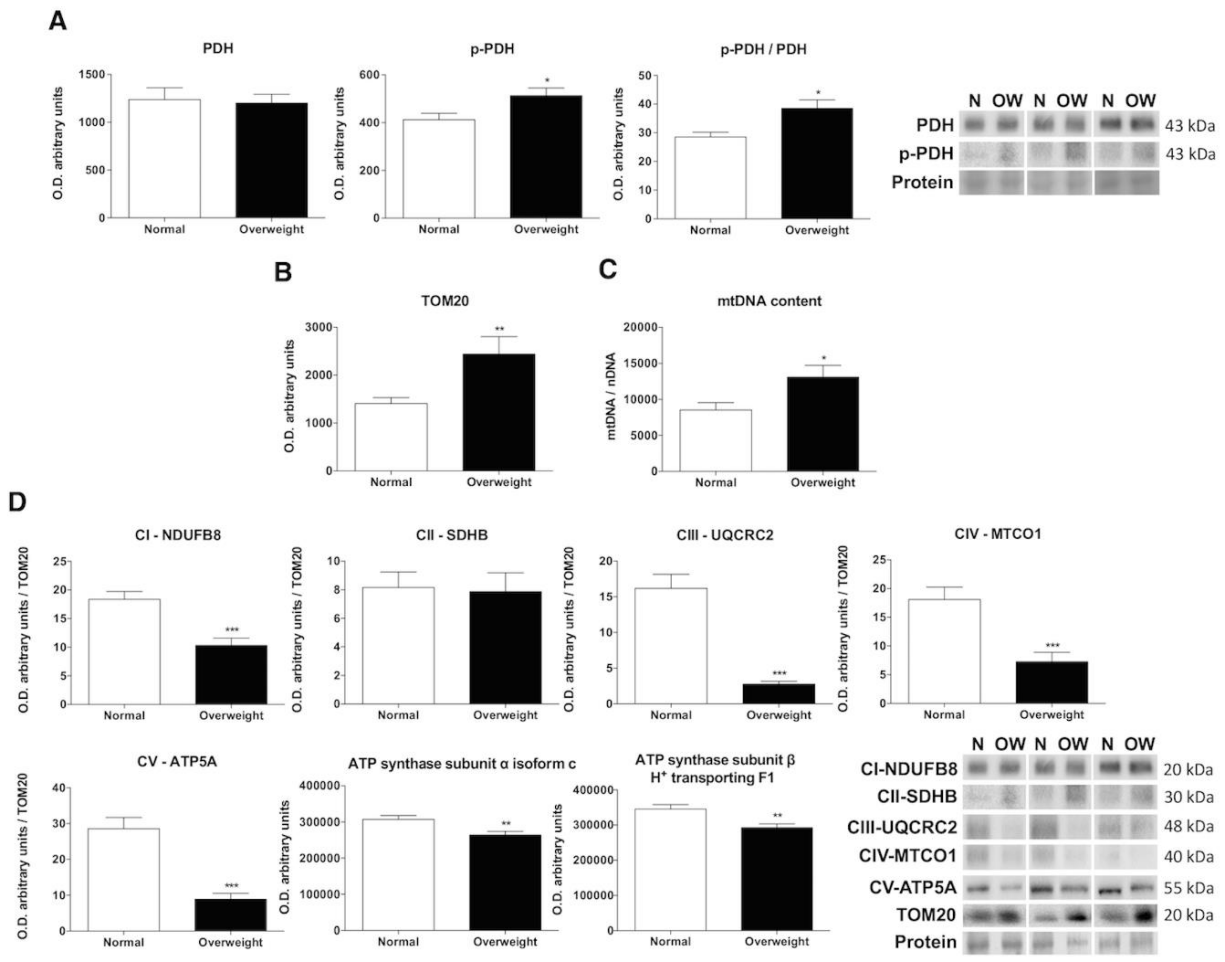
Oxidative phosphorylation profile. Laser desorption/ionization-time of flight (MALDI-TOF/TOF) mass spectrometry, RT-qPCR and western blot analysis for studying the oxidative phosphorylation pathway in muscle tissues of normal weight (N) and overweight (OW) patients. (A) Levels of proteins implied in pyruvate dehydrogenase activity regulation (pyruvate dehydrogenase (PDH), phospho-pyruvate dehydrogenase (p-PDH) and its ratio (p-PDH/PDH)). Bar charts show means of semi-quantitative optical density (O.D.) ± SEM. Representative immunoblots of PDH and p-PDH. Ponceau staining was used as a loading control (B) The amount mitochondrial content is marked by the protein levels of TOM20. Bar charts show the means of semi-quantitative optical density (O.D.) ± SEM. (C) RT-qPCR was used to confirm mitochondrial DNA (mtDNA) content. mtDNA content was compared against genomic DNA (nDNA). Data are expressed as the mean ± SEM (D) Levels of subunits from the protein complexes of the mitochondrial electron transport chain (NADH dehydrogenase (ubiquitone) 1b subcomplex 8 (NDUFB8) from complex I (CI), iron sulfur subunit (SDHB) from complex II (CII), ubiquinolcytochrome c reductase core protein II (UQCRC2) subunit from complex III (CIII), cytochrome c oxidase subunit I (MTCO1) from complex IV (CIV), and ATP synthase subunit α (ATP5A) from complex V (CV), isoform c of the ATP synthase subunit α from complex V and the H^+^ transporting F1 complex subunit β from complex V). Bar charts show the means of semi-quantitative optical density (O.D.) ± SEM. Representative immunoblots of subunits from the protein complexes of the mitochondrial electron transport chain (CI, CII, CIII, CIV and CV). Ponceau staining was used as a loading control and TOM20 as a mitochondrial marker **, P < 0.01; ***, P < 0.001.

### Skeletal muscle from overweight aged people displays altered cellular and mitochondrial bioenergetics

To examine the three-skeletal muscle metabolic energy systems, markers for the phosphagen energy pathway, glycolysis and mitochondrial status were investigated. The first pathway triggered to regenerate ATP levels is the phosphagen energy system measured by the creatine kinase (CK) and adenylate kinase enzymatic markers. These proteins were obtained from band 7 and 10 from the experimental block MALDI-TOF, and their expression levels were significantly decreased in overweight patients (p<0.05, p<0.01) (Fig. 1A). When energy requirements are increased, ATP regeneration is increasingly derived from blood glucose and muscle glycogen stores, which is mediated by glycolysis. Band 2 of the SDS-PAGE gel exhibited significantly lower levels of glycogen phosphorylase in samples derived from overweight subjects (p<0.001) (Fig. 1B). Accordingly, overweight decreases glycolysis by downregulating several major sensitive biochemical controls of both phases of glycolysis, as demonstrated by the lower levels of fructose 1,6-bisphosphate aldolase A (p<0.01), glyceraldehyde 3-phosphate dehydrogenase (p<0.05) and pyruvate kinase (p<0.001) obtained from bands 8, 9 and 3 derived from overweight subjects. However, no differences were found in β-enolase protein expression obtained from band 6 (Fig. 1C). As pyruvate kinase catalyzes the final step in glycolysis and it is one of the main enzymes that play a major role in regulating cell metabolism, its activity was determined. Consistent with protein analysis, pyruvate kinase activity was significantly lower in overweight aged people (p<0.01) (Fig. 1D).

**Figure 3.**
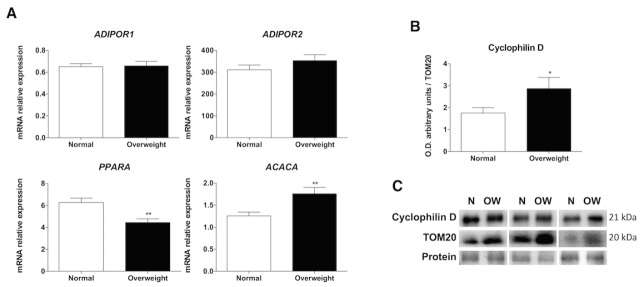
Fatty acid β-oxidation and fatty acid synthesis pathways. RT-qPCR and western blot analysis for studying the fatty acid β-oxidation and synthesis pathways in muscle tissues of normal weight (N) and overweight (OW) patients. (A) Relative mRNA expression of genes implicated in fatty acid β-oxidation (*ADIPOR1* , *ADIPOR2* and peroxisome proliferator-activated receptor alpha (*PPARA* )) and in fatty acid synthesis (acetyl-Coa carboxylase (*ACACA* )). Bar charts show the means of mRNA relative expression ± SEM. (B) Cyclophilin D protein levels. Bar charts show means of semi-quantitative optical density (O.D.) ± SEM. (C) A representative immunoblot of cyclophilin D. Ponceau staining was used as a loading control and TOM20 as a mitochondrial marker *, P < 0.05; **, P < 0.01.

Pyruvate dehydrogenase (PDH) is the one-way link between glycolysis and the oxidative pathway transforming pyruvate into acetyl-CoA. As well as, PDH is controlled by the phosphorylation at Ser293 of E1-alpha subunit that blocks its activity. Overweight people did not show variation in the total PDH protein levels. However, the levels of p-PDH and therefore p-PDH/PDH rate were higher in overweight aged people indicating a greater inactivity of this pathway in comparison to normal-weight patients (p<0.01) (Fig. 2A). The resynthesis of ATP by mitochondrial oxidative phosphorylation was evaluated by measuring mitochondrial mass, the expression of electron transport chain complexes and the main biomarkers involved in the oxidation and degradation of fatty acids. The relative abundance of TOM20, the mitochondrial mass marker, was significantly greater in the overweight group (p<0.01) (Fig. 2B). This data was confirmed by mtDNA content (p<0.01) (Fig. 2C). In contrast, immunoblotting revealed that subunits of the mitochondrial complexes I, III, IV and V were significantly reduced (p<0.001) in muscle from the overweight patients, but there were no between group differences in the content of complex II. Supporting this data, bands 4 and 5 of the SDS-PAGE gel analyzed by MALDI-TOF demonstrated that overweight patients also expressed significantly decreased levels of mitochondrial isoform c of the ATP synthase subunit α (p<0.01) and the H^+^ transporting F1 complex β subunit from the mitochondrial ATP synthase (p<0.01) (Fig. 2D). For the oxidation and degradation of fatty acid pathways, the two experimental groups showed opposing patterns, with overweight patients exhibiting a reduction in the relative mRNA expression level of peroxisome proliferator-activated receptor alpha (*PPARA* ) (p<0.01) and an increase in the levels of acetyl-Coa carboxylase (*ACACA* ) (p<0.01); however, the levels of the adiponectin receptors, *ADIPOR1* and *ADIPOR2* , did not present any variation (Fig. 3A). Moreover, the protein levels of cyclophilin D, which is recognized as a constituent of the mitochondrial permeability transition pore (mPTP), were higher in overweight aged people that may be induced by impaired fatty acid β-oxidation (Fig. 3B).

**Figure 4.**
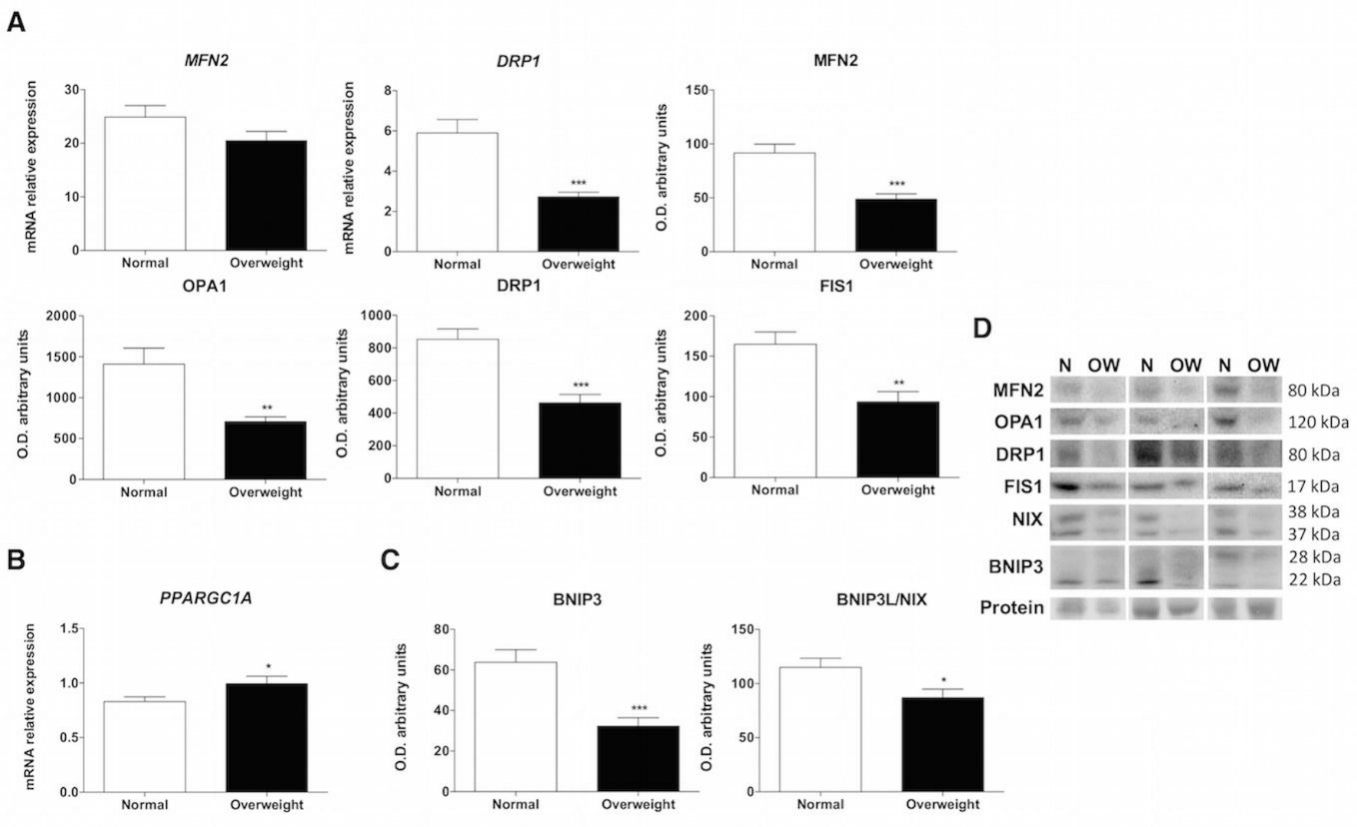
Characterization of mitochondrial dynamics, biogenesis and mitophagy pathways. RT-qPCR and western blot analysis for studying mitochondrial dynamics, biogenesis and mitophagy in muscle tissues of normal weight (N) and overweight (OW) patients. (A) Levels of mRNA and proteins involved in mitochondrial dynamics. (mRNA levels of mitofusin 2 (*MFN 2* ) and dynamin-related protein 1 (*DRP1* ) and protein levels of mitofusin 2 (MFN 2), optin atrophy protein 1 (OPA1), dynamin-related protein 1 (DRP1) and mitochondrial fission 1 protein (FIS1). Bar charts show means of mRNA relative expression and semi-quantitative optical density (O.D.) ± SEM. (B) Levels of peroxisome proliferator-activated receptor-γ coactivator 1α (*PPARGC1A* ) involved in mitochondrial biogenesis. Bar charts show means of mRNA relative expression ± SEM. (C) Levels of proteins involved in mitophagy (Bcl-2 nineteen-kilodalton interacting protein 3 (BNIP3) and Bcl-2 nineteen-kilodalton interacting protein 3-like (BNIP3L/NIX)). Bar charts show means of semi-quantitative optical density (O.D.) ± SEM. (D) Representative immunoblots of mitochondrial dynamics and mitophagy markers. Ponceau staining was used as a loading control *, P < 0.05; **, P < 0.01; ***, P < 0.001.

### Skeletal muscle from overweight aged people presents changes in mitochondrial dynamics

Mitochondria are dynamic organelles that undergo fusion and fission to maximize mitochondrial bioenergetics under stressful conditions. Mitofusin 2 (MFN2) and optin atrophy protein 1 (OPA1) are the main proteins involved in outer and inner mitochondrial membrane fusion, respectively. During fission, the dynamin-related protein 1 (DRP1) is recruited from the cytosol to the outer mitochondrial membrane, where it assembles with the mitochondrial fission 1 protein (FIS1) [35]. The results showed no changes in the mRNA levels of *MFN2* in overweight patients, but a significant decrease was detected in the *DRP1* levels (p<0.001). Conversely, the dynamic status measured by western blot analysis in whole tissue extracts revealed differences resulting from a primary deficiency in the mitochondrial fusion proteins MFN2 (p<0.001) and OPA1 (p<0.01) in overweight patients. Fusion mechanism was also affected by overweight that decreased the protein levels of DRP1 (p<0.001) and FIS1 (p<0.01) (Fig. 4A). Thus, overweight seemed to affect the regulation of mitochondrial dynamics during aging decreasing both cellular processes.

### Skeletal muscle from overweight aged people shows an imbalance between mitochondrial biogenesis and mitophagy processes

The coordination of mitochondrial biogenesis and mitophagy is a key process to maintain mitochondrial bioenergetics. An imbalance between these molecular mechanisms leads to cellular dysfunction. RT-qPCR analysis demonstrated greater mitochondrial biogenesis in overweight individuals, as measured by comparing the mRNA levels of the peroxisomal proliferator-activated receptor coactivator 1 α (*PPARGC1A* ) (p<0.05) (Fig. 4B), accompanied by a lower mitophagy response, as analyzed by comparing the protein levels of the Bcl-2 nineteen-kilodalton interacting protein 3 (BNIP3) (p<0.001) and Bcl-2 nineteen-kilodalton interacting protein 3-like (BNIP3L/NIX) (p<0.05)(Fig. 4C).

**Figure 5.**
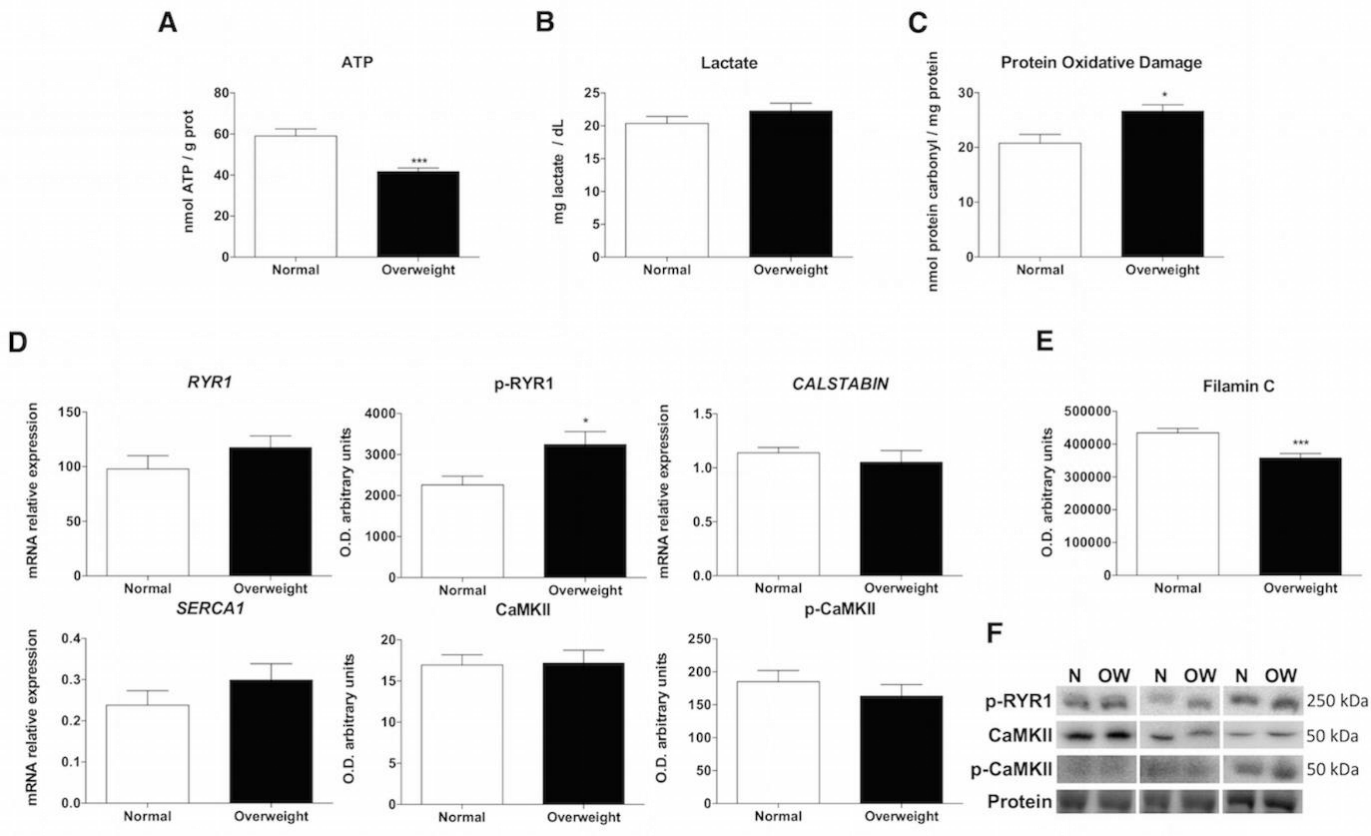
Skeletal muscle ATP production, plasma lactate content, oxidative protein damage and characterization of excitation-contraction coupling mechanism. Luminometric and spectrophotometric analysis for ATP and oxidative protein damage in muscle tissue, lactate in plasma and RT-qPCR, western blot and laser desorption/ionization-time of flight (MALDI-TOF/TOF) mass spectrometry analysis for studying muscle contraction and integrity in muscle tissues of normal weight (N) and overweight (OW) patients. (A) Levels of ATP in skeletal muscle. The ATP content is presented as nmol ATP/g protein. Data are expressed as the mean ± SEM. (B) Levels of lactate in plasma. The lactate content is presented as mg lactate/dL. Data are expressed as the mean ± SEM. (C) Levels of protein oxidative damage in skeletal muscle. The protein oxidative damage is presented as nmol protein carbonyl/mg protein. Data are expressed as the mean ± SEM. (D) Levels of mRNA and proteins required for excitation-contraction coupling (Ryanodine receptor 1 (*RYR1* ), phospho-RYR1 (p-RYR1), *CALSTABIN* , sarco/endoplasmic reticulum Ca^2+^-ATPase 1 (*SERCA1* ), Ca^2+^/Calmodulin dependent protein kinase II (CaMKII), phospho-CaMKII (p-CaMKII)). Bar charts show the means of mRNA relative expression and semi-quantitative optical density (O.D.) ± SEM. (E) Levels of filamin C protein involved in the maintenance of structural integrity. Bar charts show the means of semi-quantitative optical density (O.D.) ± SEM. (F) Representative immunoblots of muscle contraction markers. Ponceau staining was used as a loading control. *, P < 0.05; ***, P < 0.001.

### Skeletal muscle from overweight aged people leads to an insufficient anaerobic cellular respiration

In view of the previous results, ATP production was also studied, and a significant drop of ATP content was showed in overweight subjects (p<0.001) (Fig. 5A). When the production of pyruvate exceeds the capacity of mitochondrial oxidative phosphorylation, pyruvate is removed from the cytosol by triggering acid lactic fermentation in order to prevent the inhibition of glycolysis. Thus, lactate production was measured. A non-significant upward trend was observed in the plasma of overweight patients (Fig. 5B). It is noteworthy that the lactate levels obtained in both experimental groups exceeded the normal range (5.7-20 mg/dL) because aging is already promoting this metabolic pathway. Since both groups were similar in age, all differences obtained between them were due only to differences in weight.

### Skeletal muscle from overweight aged people displays excitation-contraction uncoupling

The observed energy capacity alterations could have a direct impact on muscle contraction. Therefore, a set of studies was performed to evaluate this process. In western blots, p-RYR1 was significantly increased in overweight patients. There were no variations in the mRNA levels of *CALSTABIN* and sarco/endoplasmic reticulum Ca^2+^-ATPase 1 (*SERCA1* ) or in the protein level and phosphorylation status of CaMKII (Fig. 5C). Nonetheless, filamin C protein, involved in the maintenance of skeletal muscle integrity and obtained from band 1 in the MALDI-TOF analysis, showed a significant decrease in expression in overweight older subjects (Fig. 5D).

## DISCUSSION

Aging attenuates the well-known metabolic plasticity [36] that allows cells to respond to various stimuli, including nutritional states [37]. Chronic diseases, such as obesity, further reduce plasticity. However, the reasons for the loss of plasticity induced by obesity during aging are not known, even when these are essential to the independence of these subjects. Hence, we analyzed the three-major energy metabolic systems that produce ATP in normal-weight and overweight patients.

Phosphagen system, also known as the phosphocreatine system, is essential for maintaining energetic homeostasis in tissues that have fluctuating energy requirements such as skeletal muscle [38]. Interestingly, most of functional enzymatic changes of this pathway occurred by middle age resulting in a decrease in its activity [39]. However, overweight induced a greater decline in the enzymatic levels of CK in an aged population. Therefore, muscle fibers of overweight people are not able to efficiently trigger this pathway to obtain age-specific levels for energy demands in situations requiring punctual high-energy expenditure. Consistent with this finding, lower levels of the adenylate kinase enzyme, a potent allosteric activator of glycogenolysis and glycolysis, was observed. Of the glycolytic biomarkers studied, the protein and activity levels of pyruvate kinase enzyme were the most notable for its reduction in overweight aged group. Some studies have demonstrated that senescent muscle has a drastic effect on glycolytic enzymes [40, 41], especially reducing pyruvate kinase expression [41, 42]. Our study demonstrates that the downregulation of pyruvate kinase activity described in aged skeletal muscle is aggravated by excess weight. The entry of glycolytic products into the Krebs cycle to promote oxidative phosphorylation is controlled by PDH. Aging does not disturb PDH activity, but it is greatly affected under fed condition [43] supposing an important metabolic remodeling in obesity. This study demonstrates that overweight decreases the PDH activity and may aggravate the mitochondrial respiratory dysfunction caused by aging [44]. In fact, strong protein expression reduction of complex I, III, IV and ATP synthase subunits in elderly overweight patients appear to indicate that the function and efficiency of oxidative phosphorylation might be affected. However, overweight aged people-maintained CII-SDHB protein levels. Complex II activity is triggered as a compensatory process to support mitochondrial metabolism, when complex I capacity is compromised [45]. This fact suggests that respiratory-chain complex I is uncoupled, and the mitochondrial energy requirements may be achieved in overweight patients via complex II activity. However, complex II, that is the unique common component of Krebs cycle and the ETC, is directly activated by metabolic sensors such us PDH [46]. Thus, the inactivation of PDH activity by overweight condition indicates that complex II could not be able to restore mitochondrial bioenergetics. To further investigate the relationship between overweight and mitochondrial function in skeletal muscle tissue from aged people, we also analyzed fatty acid β-oxidation and synthesis pathways. An increase in total energy expenditure and a reduction in body fat mass as well as skeletal muscle triglyceride levels have been observed in *ACACA* knockout mice, thus highlighting the role of *ACACA* in diet-induced obesity [47]. Our study showed that overweight people not only did not maintain the fatty acid β-oxidation displayed by *PPARA* pathway but also exhibited enhanced fatty acid synthesis, as reflected by the increased *ACACA* levels, thus providing further evidence of energy homeostasis alteration at such an early stage of obesity. Fatty acid accumulation has a prominent effect on mitochondrial energy coupling by inducing respiratory inhibition and altered mPTP state [48]. Moreover, a deficiency of cyclophilin D levels, which is a critical regulator of the mPTP, is related to fat mass loss due to the trigger of mitochondrial oxidative metabolism and fat oxidation [49]. A recent study in Tfam knockout mice and patients with mitochondrial myopathy proved that the downregulation of cyclophilin D is essential for counteracting mitochondrial skeletal muscle dysfunction [50]. Accordingly, in our study, overweight patients presented higher levels of cyclophilin D suggesting again an altered mitochondrial function.

Mitochondrial dynamics, the balance of fission and fusion, are fundamental to the regulation of mitochondrial distribution as well as bioenergetics. Nutrient excess stimulates mitochondrial fission and inhibits fusion in depolarized and uncoupled mitochondria, usually observed in age-related diseases [51-53]. We found that overweight subjects not only exhibited lower levels of DRP1 and FIS1 proteins that promote fission, but also lower levels of MFN and OPA1 which are involved in the outer and inner mitochondrial fusion, respectively. Altogether, overweight induces important changes in the molecular machinery of mitochondrial dynamics marked by a depletion of both cellular processes in whole skeletal muscle extracts. Thus, the downregulation of mitochondrial fusion machinery, that is important for the maintenance of mitochondrial respiration under pathological conditions, also indicates a mitochondrial dysfunction. Moreover, an imbalance in mitochondrial dynamics skewed towards increased fission implies an accumulation of damaged mitochondria that triggers the mitophagy pathway [54]. However, myopathies and other age-associated disorders underlie the development of an imbalance resulting from mitochondrial proliferation and a lack of degradation processes [55, 56]. Mitophagy is an energy-dependent process; so impaired energy production in the overweight group may have impaired mitochondrial selective autophagy. A previous study from our laboratory using the same population cohort also demonstrated that overweight lead to an accumulation of autophagosomes, which were not properly degraded and resulted in an accumulation of damage [26]. Both facts linked to an increase in mitochondrial biogenesis lead to progressive mitochondrial accumulation, which is supported by the increased levels of mtDNA content and the TOM20 mitochondrial marker. Moreover, the combination of mitochondrial dysfunction and defective autophagy could contribute to increased age-related pathologies [57] and aggravate sarcopenia and physical frailty [58]. Therefore, the dysfunctional mitochondria accumulation in the muscle of our overweight cohort seems to be determinant for developing sarcopenia and frailty.

**Figure 6.**
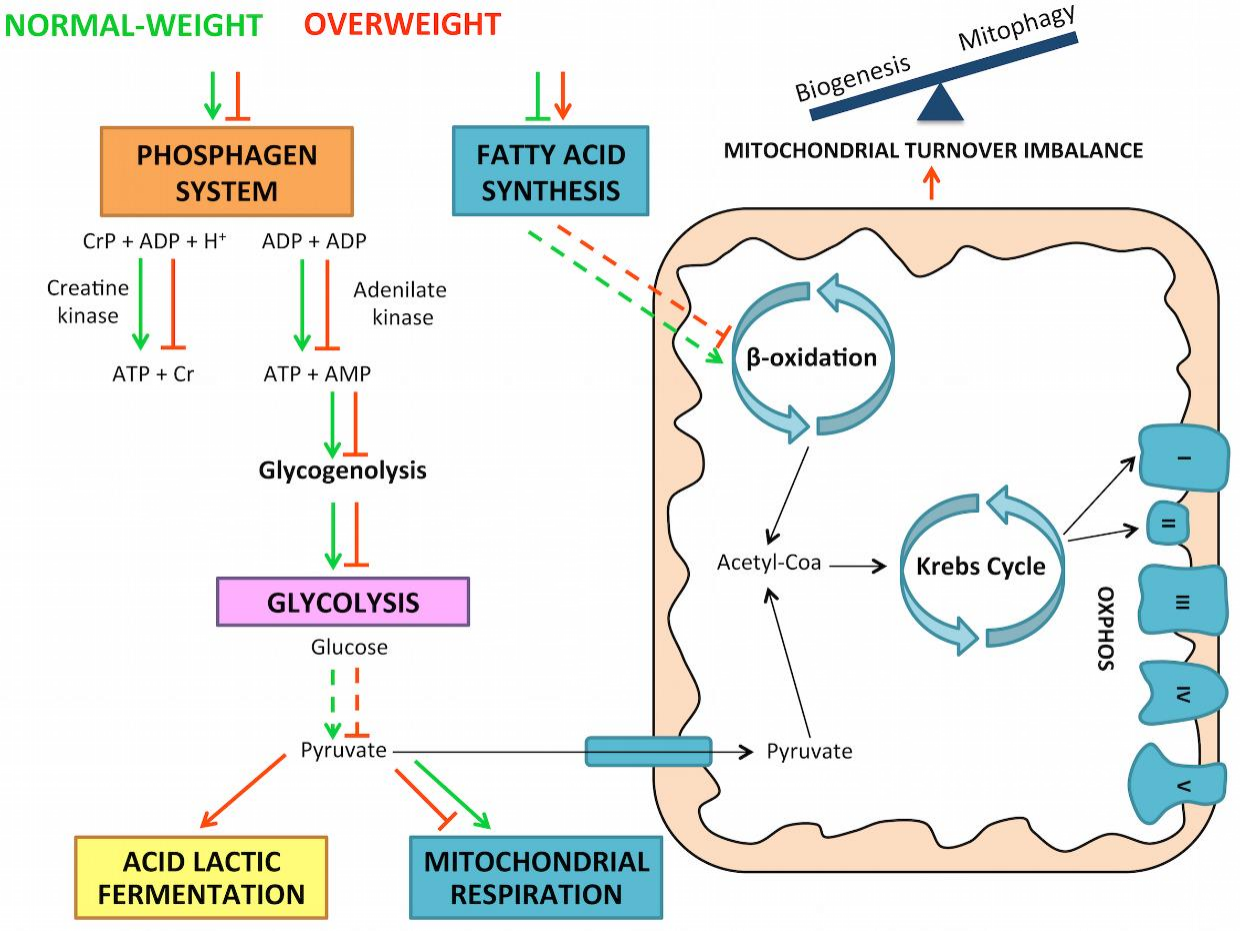
Global effect of overweight during aging on energy metabolic systems. Overweight induces a defective phosphagen, glycolysis and oxidative phosphorylation metabolic pathways during aging, resulting in a switch from oxidative to lactic acid fermentation metabolism.

In the overweight elderly, a combination of mitochondrial dysfunction and a lack of expression of the major energy-producing cellular biomarkers involved in the phosphagen system, glycolysis and mitochondrial respiration led to a decline in the ATP levels. Based on these results, the lactic acid fermentation pathway was also evaluated revealing an upward trend in the concentration of plasma lactate. This finding suggests that overweight induces a metabolic switch from oxidative to fermentation metabolism. In this context, lactate production was not due to glycolytic stimulation but resulted from pyruvate shunting toward fermentation, which permits continued ATP production under mitochondrial dysfunction (Fig. 6). In spite of the remarkable plasticity in energy production exhibited by skeletal muscle tissue, overweight aged subjects are not able to restore basal ATP to the same levels seen in normal-weight aged subjects. Furthermore, altered energy production, especially by the perturbation of the mitochondrial respiratory chain leads to oxidative stress production [59]. Likewise, a hallmark of aging is the accumulation of oxidatively modified proteins, which in turn impact in energy metabolism and muscle contraction contributing to the generation of the sarcopenic phenotype [60]. This study demonstrates that overweight increases oxidative protein damage playing a critical role in aged skeletal muscle. Overweight causes early signs of impaired contractile function, such as the observed increase in RYR1 channel phosphorylation. RYR1 modifications may be the consequence of insufficient energy production and excessive mitochondria-derived reactive oxygen species (ROS) [19, 61]. Moreover, the lower filamin C levels in the overweight aged patients provides further evidence for a decline in skeletal muscle structural integrity. Thus, as a consequence of the energy production impairment, overweight decreases structural muscle integrity that anticipates the onset of dependence. This research may be crucial for diagnosing and confronting both overweight and obesity during aging.

## Supplementary Materials

The Supplemenantry data can be found online at: www.aginganddisease.org/EN/10.14336/AD.2018.0430
